# Capsulized faecal microbiota transplantation ameliorates post-weaning diarrhoea by modulating the gut microbiota in piglets

**DOI:** 10.1186/s13567-020-00779-9

**Published:** 2020-04-16

**Authors:** Wenjie Tang, Daiwen Chen, Bing Yu, Jun He, Zhiqing Huang, Ping Zheng, Xiangbing Mao, Yuheng Luo, Junqiu Luo, Quyuan Wang, Huifen Wang, Jie Yu

**Affiliations:** Animal Nutrition Institute, Sichuan Agricultural University and Key Laboratory of Animal Disease-Resistance Nutrition, Ministry of Education of China, Chengdu, China

## Abstract

Early weaning-induced stress causes diarrhoea, thereby reducing the growth performance of piglets. Gut bacterial dysbiosis has emerged as a leading cause of post-weaning diarrhoea. The present study aimed to investigate the effect of capsulized faecal microbiota transplantation (FMT) on the gut bacterial community, immune response and gut barrier function of piglets. Thirty-two weaned barrows were randomly divided into two groups. The recipient group was inoculated orally with capsulized faecal microbiota of healthy Tibetan pigs during the whole period of the trial, while the control group was given an empty capsule. The feed-to-gain ratio, diarrhoea ratio, and histological damage score of recipient piglets were significantly decreased. FMT treatment significantly increased the colon length of piglets. Furthermore, the relative abundances of *Firmicutes*, *Euryarchaeota*, *Tenericutes*, *Lactobacillus*, and *Methanobrevibacter* in the colon of recipient piglets were increased, and the relative abundances of *Campylobacter* and *Proteobacteria* were significantly decreased compared with those in the control group. CD4^+^ lymphocytes and CD4^+^/CD8^+^ ratio in the peripheral blood of recipient piglets were significantly increased. FMT treatment increased the IL-4 and IL-10 levels and decreased the TNF-α and INF-γ levels in the colonic tissue of piglets. The recipient piglets’ mRNA expression of TLR2, TLR8, NF-κB, and iNOS was significantly regulated. In addition, FMT significantly enhanced the gene expression of ZO-1. Overall, treatment with capsulized FMT ameliorated diarrhoea in piglets, with significant effects on limiting colon inflammatory responses, downregulating the TLR signalling pathway and the gene expression of iNOS, and strengthening intestinal barrier function by modulating the constituents of the gut microbiota.

## Introduction

The mammalian digestive tract harbours a complex and dynamic microbial ecosystem mainly composed of bacteria. The gut microbiota has various roles benefiting the host, such as energy metabolism, pathogen resistance, and cellular immunity [1, 2]. Intestinal microbiota disorders can also lead to host gastrointestinal diseases such as inflammatory bowel disease, irritable bowel syndrome, and metabolic syndrome [3]. Diseases caused by intestinal microbiota dysbiosis can be improved by therapeutic modalities involving microbiota, such as faecal microbiota transplantation (FMT) [4, 5]. FMT may efficaciously transplant the gut microbial community from a healthy donor stool to the recipient. FMT has been developed rapidly over the last few years from a crude procedure involving different routes of administration of raw stool from patient-identified donors to the use of purified and cryopreserved standardized preparations of faecal microbiota from highly selected donors [6]. FMT provides a valid therapeutic benefit after the onset of refractory recurrent *Clostridium difficile* infection by reintroducing a balanced microbiota to restore the structure and function of the gut microbial community [7]. Clinical applications of FMT have provided increasing convincing evidence that modification of the intestinal microbiota results in impressive cure rates of intestinal dysbiosis-related diseases [8].

Currently, weaning is a critical event in the pig’s life cycle that is associated with serious enteric health concerns. The disruption of the piglet intestinal microbial balance, induced by abrupt changes in the diet and environment of piglets, can predispose them to post-weaning diarrhoea [9]. Most studies focusing on the weaning transition of piglets have reported a reduction in the biodiversity of intestinal microbiota diversity [10], and such disturbances of the gut microbial ecosystem and loss of diversity at early stages of life can strikingly increase the risk of post-weaning diarrhoea and enteric infections [11]. Thus, FMT could be a potential method for intestinal microbiota re-establishment and diarrhoea amelioration in piglets. Indeed, the exploitation of a feasible protocol for FMT operation in livestock farming is ongoing. In piglet models, colonoscopic administration of FMT usually causes severe stress, especially in cases of long-term intervention. Oral preparation is preferable for piglets due to its convenience. Easy-to-handle capsules containing the donor’s faecal microbiota provide a safe and highly effective treatment for recurrent *Clostridium difficile* syndrome [12], which suggests the availability and efficiency of FMT capsules. However, the connection between FMT and the structural changes of gut microbiota is not fully illustrated, and even the possibility of capsule FMT to ameliorate the diarrhoea of piglets from weaning stress as well as the underlying mechanism is little known. Meanwhile, regarding donor selection, Tibetan pigs, which are a domestic breed in China, are unique in phenotype and physiological characteristics compared with commercial pig breeds, which endows them with increased resistance to disease [13]. Our research group previously found that pigs inoculated with the Tibetan microbiota acquired relatively strong resistance to experimental diarrhoea [14]. In the present study, we hypothesized that capsule FMT would modulate the structure of the gut microbiota, ameliorate diarrhoea caused by weaning stress, and improve immune function in recipient piglets. Weaning piglets were selected to explore the effect of capsulized Tibetan pigs’ faecal microbiota on gut microbiota, diarrhoea, growth performance, and immune traits of weaning piglets.

## Materials and methods

### Animal care, diet, and experimental design

All methods in this study were performed in accordance with the protocol outlined for the Care and Use of Laboratory Animals prepared by the Institutional Animal Care and Use Committee of Sichuan Agricultural University (SAU), and all animal protocols were approved by the Animal Care and Use Committee of SAU under permit number S20174311. Three Tibetan pigs from Sichuan Reservation Farm were used in the current research as faecal donors. The donors were similar in age, were raised from birth in the same environment and were fed the same diet without antibiotics. Routine diagnoses for specific pathogens, including viruses and parasites, were performed before the experiment to ensure that the donors met the SAU-specific pathogen-free standard for pigs [14].

A total of eight litters (4 piglets per litter) of DLY (Duroc × Landrace × Yorkshire) weaned barrows (6.55 ± 0.02 kg) of the same age (25 days) were fed in metabolic cages (1.4 m × 0.6 m × 0.8 m) with a 1-sided feeder and stainless-steel nipple drinker. The room temperature was controlled at 26 ± 1 °C. The basal diet was formulated to meet the nutrient requirements recommended by the National Research Council (NRC 2012) [15], and the ingredient composition and nutrient levels are shown in Additional file 1. Piglets were randomly allotted to two groups (*n* = 16 per group) matched for body weight and litter origin as follows: the FMT group received capsulized faecal microbiota for 21 days (full-trial period); the control group received empty capsules for 21 days; all pigs received the orally capsulized material successfully. The same diet was used for all groups throughout the process. Individual body weight (BW) was recorded after all pigs were food-deprived for 12 h on day 21, and feed consumption was recorded as the amount of feed offered daily minus the remaining quantity on the next morning during the experiment, which were used to determine the average weight gain (ADG), average daily feed intake (ADFI) and ratio of feed to gain (F/G).

### Preparation of faecal microbiota capsules and process of transplantation

Fresh faecal bacterial suspensions of healthy Tibetan pigs were prepared using the standard method as described by Hu’s research [16], and the age of the donor Tibetan pigs ranged from 80 ~ 100 days. All faecal material preparation processes were carried out in an anaerobic incubator (Thermo Scientific 1029, USA), and the room temperature was controlled at 26 °C. The suspension was immediately encapsulated in calcium alginate following Chandramouli et al. [17] with modifications. In brief, 2% w/v sodium alginate only; 4% w/v skim milk only; 2% w/v sucrose only; 5% v/v mannitol only; 2% v/v glycerol only; 80% v/v faecal bacteria suspension only. After mixing the above-mentioned sodium alginate, cryoprotectants, and faecal bacteria suspension evenly, the final product was hardened for a period of 30 min to form capsules in calcium chloride (1 M). The total dose was 1.7 × 10^11^ cells/g. Cell numbers were determined by the method of the fluorescence spectrum of bacterially bound acridine orange [18]. The capsules were secondarily sealed (manually operated) by using size 0 enteric and acid-resistant hypromellose capsules (DRCaps, Capsugel). The finished products were stored frozen at −80 °C, and the final concentration of the capsule was 8.9 × 10^10^ cells/capsule. The stability of capsules in an acidic environment mimicking the stomach was tested internally by evaluating trypan blue-filled capsules [12]. At 37 °C and a pH of 2.5, the capsules were stable for 185 min before dye was released. Two hours prior to administration, the capsules were transferred to −20 °C. Piglets in both groups (FMT group, capsulized faecal microbiota; control group, vacant capsule) were administered 3 capsules daily in the morning for the full experimental period. The recipient piglets were allowed only water for 2 h before taking the capsules. Then, the gavage method was used to place the capsule at the back of the piglet’s tongue with the irrigating water.

### Necropsy procedures and diarrhoea evaluation

For the Tibetan pigs, spontaneously excreted faecal samples were collected from 3 Tibetan pigs within the same breed after 12 h of fasting. Then, samples were stored at −80 °C prior to DNA extraction for microbial structure analysis. All pigs were food-deprived for 12 h before final necropsy. The blood of 32 DLY piglets was gathered through the jugular vein, and 2 mL of whole blood was added to an EDTA-coated tube for the lymphocyte subtype assay. Next, the piglets were euthanized by intravenous injection of chlorpromazine hydrochloride (3 mg/kg body weight). The abdomen was immediately opened. The macroscopic view of the colon was observed, and the colon length from both groups was measured. The mucosal samples from the colon were harvested by scraping with a sterile glass microscope slide, rapidly frozen in liquid nitrogen and stored at −80 °C for gene expression and cytokine determination. The colon segments (distal section of the colon) were sampled and fixed into 10% buffered formalin at 4 °C for morphometric analysis and histological injury score. The morphometric analysis was carried out by staining with eosin and haematoxylin. The specimens were examined by an Olympus CK 40 microscope at 100× magnification. The crypt depth was measured and analysed using Image-pro plus 6.0 (Media Cybernetics, Inc., Rockville, MD, USA). Scores ranged from 0 to 12, which represents the sum of scores for severity of damage, inflammation, and bleeding (Additional file 2). The colonic digesta were collected into sterile containers and stored at −80 °C, pending microbial analysis. The body weight and stool consistency (0, normal; 1, pasty; 2, semiliquid; and 3, liquid) were reported daily. Pigs showing scores of 2 or 3 were considered to have diarrhoea. The diarrhoea ratio and index of each piglet were calculated as follows:

$${\text{Diarrhoea raito (\% )}} = {\text{A/}}21 \times 100\%$$

where A = total number of days with diarrhoea. 21 = days of the whole experimental period.

The diarrhoea index was calculated as follows:

$${\text{Diarrhoea index}} = {\text{B/(21}}\; \times \;3)\; \times \;100{\text{\% }}$$

where B = total score number of every pig. 21 × 3 = the full score of the whole experimental period.

### Colon cytokine assay

Colonic tissue (80 mg) was added to ice-cold PBS solution at 4 °C and pulverized using an ultrasonic cell disruption system (Sicentz-IID, Scientz, Ningbo, China) and then centrifuged at 3500 *g* for 10 min at 4 °C. The levels of TNF-α, IFN-γ, IL-1β, IL-4, IL-6, IL-10, IL-12, and IL-13 in the colon tissue supernatant were measured by ELISA according to the manufacturer’s instructions (R&D Systems, Minneapolis, MN, USA).

### Lymphocyte subtype assay

Lymphocyte subtypes were analysed by a flow cytometer (FACSVerse, BD Biosciences, USA). A premixed cocktail of monoclonal antibodies (CD3-FITC, CD4-Percp and CD8-PE (BD Biosciences, USA)) were added into a 12 mm × 65 mm tube with 100 μL EDTA blood samples, and then the mixtures were gently mixed in the dark for 30 min. Thereafter, 2 mL RBC lysing solution (BD Biosciences, USA) was added to the tube and incubated for another 10 min. The mixture was centrifuged at 500 *g* for 5 min and washed with 2 mL of PBS. The cell pellets were resuspended in 1 mL of 1% paraformaldehyde in PBS and measured by a flow cytometer. The percentage of T-helper cells (CD3^+^CD4^+^) and cytotoxic T-cells (CD3^+^CD8^+^) were analysed by FlowJo software (FlowJo LLC, Ashland, OR, USA).

### Total RNA isolation and real-time quantitative PCR

The RNA of colon tissue samples was extracted by using RNAzol (Invitrogen) following the manufacturer’s instructions. The materials and methods of quantifying the purity and integrity of the RNA were the same as previously described [14]. The RNA samples were reverse transcribed into cDNA using the PrimeScript™ RT reagent kit (Takara) according to the manufacturer’s instructions. The gene-specific primers used in the present study are shown in Additional file 3 and were commercially synthesized by TaKaRa Biotechnology. The relative mRNA expression was measured by real-time quantitative PCR with the CFX96 Real-Time PCR Detection System (Bio-Rad) as previously described [19]. The relative gene expression ratio of the target gene to the reference gene (β-actin) was calculated according to the ΔΔ Ct method as described previously [20].

### DNA extraction and 16S rRNA amplicon sequencing

Microbial genomic DNA was extracted from faecal samples of Tibetan pigs and colonic digesta of DLY piglets using the QIAamp DNA Stool Mini Kit (Qiagen, GmbH Hilden, Germany) according to the provided manual. The purity and integrity of the extracted genomic DNA were quantified as previously described [14]. According to the concentration, DNA was diluted to 1 ng/µL using sterile water, and 10 µL of DNA dilution was used for sequencing. Sequencing was performed at Novogene Bioinformatics Technology Co. Ltd., Beijing, China. The V3–V4 hypervariant region of the bacterial 16S rRNA gene was amplified by using the 341F primer (5′-CCTAYGGGRBGCASCAG-3′) and 806R primer (5′-GGACTACNNGGGTATCTAAT-3′) (initial denaturation at 98 °C for 1 min, followed by 30 cycles of 98 °C for 10 s, 50 °C for 30 s, 72 °C for 30 s, and 72 °C for 5 min). All PCRs were carried out by using Phusion^®^ High-Fidelity PCR Master Mix (New England Biolabs). Products were purified with the GeneJET™ Gel Extraction Kit (Thermo Scientific) to remove the unspecific products. Sequencing libraries were generated using the Ion Plus Fragment Library Kit 48 rxns (Thermo Scientific) following the manufacturer’s recommendations. The library quality was assessed on the Qubit@ 2.0 Fluorometer (Thermo Scientific). Finally, the library was sequenced on an Ion S5™ XL platform, and 400 bp/600 bp single-end reads were generated.

### Processing of sequencing data

First, the raw data were preprocessed to eliminate adapter pollution and low quality to obtain clean reads. The paired-end clean reads with overlaps were merged to tags by Connecting Overlapped Pair-End (COPE) software [21, 22]. Bacterial tags were clustered into operational taxonomic units (OTUs) based on 97% sequence similarity by scripts of UCHIME software [23, 24]. Sequence analysis was performed by Uparse software (Uparse v7.0. 1001) [25]. Sequences with ≥97% similarity were assigned to the same OTUs. Then, the Silva Database [22] was used based on the Mothur algorithm to annotate taxonomic information for each representative sequence. Subsequent analyses of alpha diversity and beta diversity were performed based on this output normalized data. PCoA (principal coordinates analysis) analysis was performed using the WGCNA package, stat packages and ggplot2 package in R software (Version 2.15.3). A heatmap was visualized using R software, and log 10 transformation was applied to the bacterial relative abundance data matrix.

### Statistics analysis

Data were analysed with SPSS (Version 21.0; IBM, USA) with each pig as the experimental unit. The results are presented as the mean and SEM. Statistical differences between treatments were determined by the unpaired *t* test. Metastats analysis was used to identify the differentially abundant taxa between groups [27]. Statistical comparison of PCoA analysis was assessed through analysis of molecular variance (AMOVA). For significance determination, *P* < 0.05 was considered significant, whereas *P* < 0.10 was considered a tendency.

## Results

### Growth performance, diarrhoea index and diarrhoea ratio

**Figure 1:**
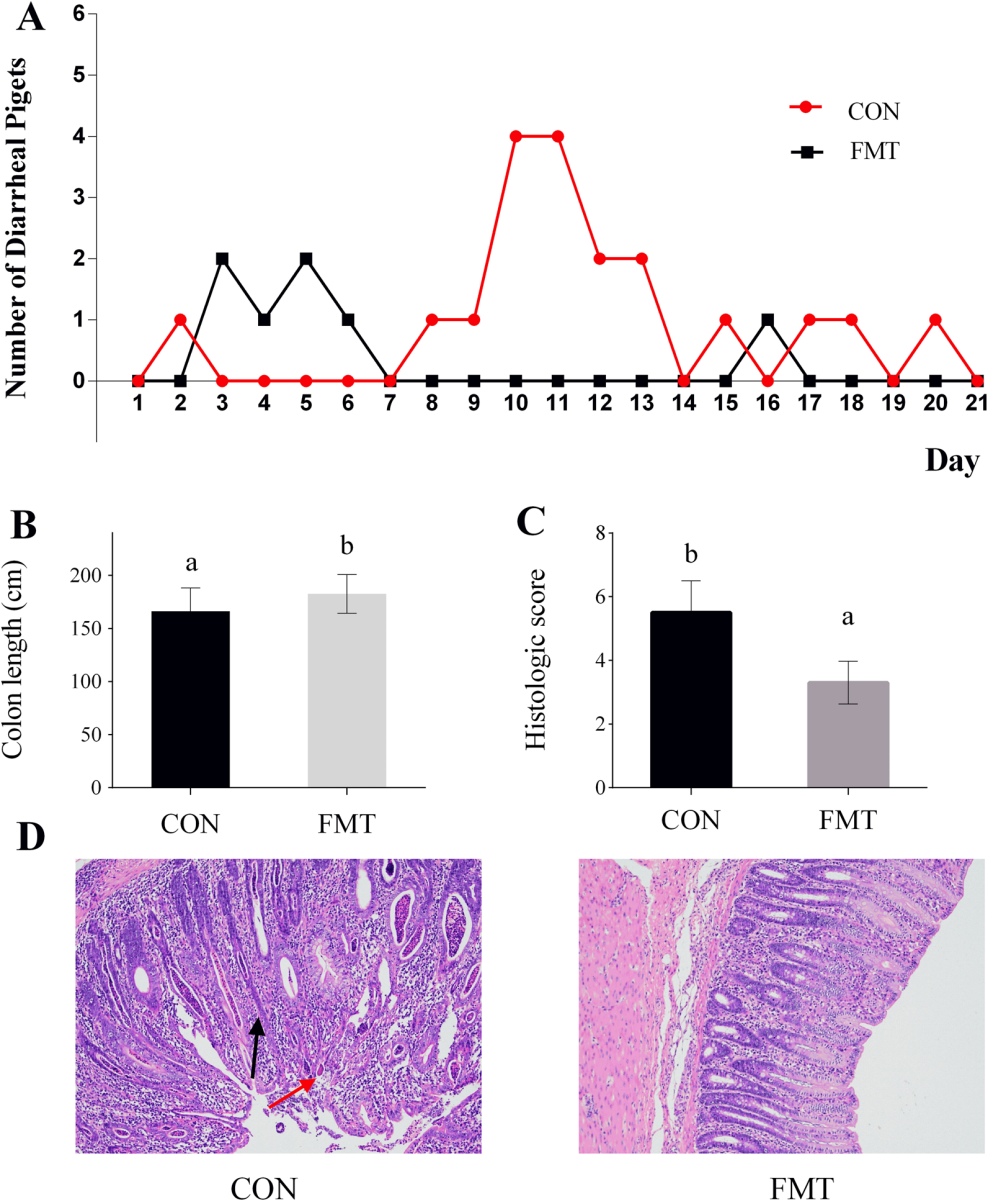
**The effects of capsulized FMT on the number of diarrhoeal piglets and colonic measurement. A** The number of diarrhoeal piglets in the FMT group was significantly less than that in the control group. **B** Colon length was increased after the capsulized FMT treatment of piglets; **C** piglets treated with capsulized FMT had a lower histological score than piglets in the control group; **D** serial sections of colon tissues were stained with H&E, original magnification 100× (red arrows indicate colon mucosal bleeding, black arrows indicate inflammatory cell infiltration). CON: control group. FMT: faecal microbiota transplantation. The data are expressed as the mean ± SEM. a, b represents significant differences between two groups (*n* = 16 per group for **A** and **B**; *n* = 10 per group for **C**).

The growth performance of piglets is shown in Table 1. There were no differences in ADFI, ADG or final BW between recipient and control piglets (*P *> 0.05). Compared with control piglets, the F/G of recipient piglets was significantly decreased (*P* < 0.05). The diarrhoea index (Table 2) of the recipient piglets was lower than that of the control piglets (*P* < 0.01). The diarrhoea ratio (Table 1) of the recipient piglets was significantly decreased compared with that of the control piglets (*P* < 0.05), and the daily number of diarrhoeal piglets in each group is presented in Figure 1A. The number of diarrhoeal piglets in the recipient group was less than that in the control group. In addition, more pigs exhibited diarrhoea episodes in the control group than in the recipient group, according to the diarrhoea score (Additional file 4).

**Table 1:** Effects of faecal microbial capsules on growth performance and diarrhoeal occurrence in piglets

Items	FMT treatment		SEM	*P* value
	CON	FMT		
Growth performance				
Initial BW (kg)	6.55	6.55	0.019	1.000
Final BW (kg)	11.47	11.91	0.139	0.118
ADFI (g/day)	418.33	426.04	8.440	0.655
ADG (g/day)	234.70	255.41	6.556	0.116
F/G (g/g)	1.81^b^	1.67^a^	0.033	0.034
Diarrhoeal occurrence				
Diarrhoea index (%)	12.20^b^	5.95^a^	1.218	< 0.01
Diarrhoea ratio (%)	5.65^b^	2.08^a^	0.893	0.043

*n* = 16. CON: control group, FMT: faecal microbial transplantation group, ADG: average weight gain, ADFI: average daily feed intake, F/G: the ratio of feed to gain.

^a,b^ In the same row, values with different letter superscripts mean significant difference.

**Table 2:** Effects of faecal microbial capsules on cytokine levels in the colon tissue of piglets

Items (pg/mL)	FMT treatment		SEM	*P* value
	CON	FMT		
IL-1β	13.21	12.52	0.524	0.521
IL-4	21.61^a^	25.61^b^	0.702	< 0.01
IL-6	304.78	289.71	7.556	0.328
IL-8	152.69	160.77	2.994	0.182
IL-10	64.46^a^	72.56^b^	1.673	0.012
IL-13	26.44	27.97	0.609	0.215
TNF-α	288.76^b^	192.03^a^	18.564	< 0.01
IFN-α	33.35^b^	30.35^a^	0.763	0.047

*n* = 14. CON: control group, FMT: faecal microbial transplantation group.

^a,b^ In the same row, values with different letter superscripts mean significant difference.

### Colon length and histological measurement

The results of the number of diarrhoeal piglets in each group, colon length and histological measurement are shown in Figure 1. The number of diarrhoeal piglets in the control group was greater than that in the FMT group. The colon length of control piglets was evidently shorter (*P* < 0.05) than that of recipient piglets (Figure 1B). The colons from the FMT group had lower histological damage scores (Figure 1C) than those from the control group (*P* < 0.05). In addition, the colons from the FMT group showed an intact morphology with complete colonic crypts. However, histopathological evaluation of the colons revealed disruptions of the epithelial layer and inflammatory cells in the submucosa of the control group (Figure 1D).

### Colon tissue cytokines

Faecal microbial capsule increased the IL-4 and IL-10 levels in the colon of weaned piglets (*P* < 0.05, Table 2). However, compared with control piglets, TNF-α and INF-γ levels of recipient piglets were significantly decreased (*P* < 0.05). There were no significant changes in IL-1β, IL-6, IL-8, or IL-13 in the colon tissue (*P* > 0.05).

### Lymphocyte subpopulation

**Figure 2:**
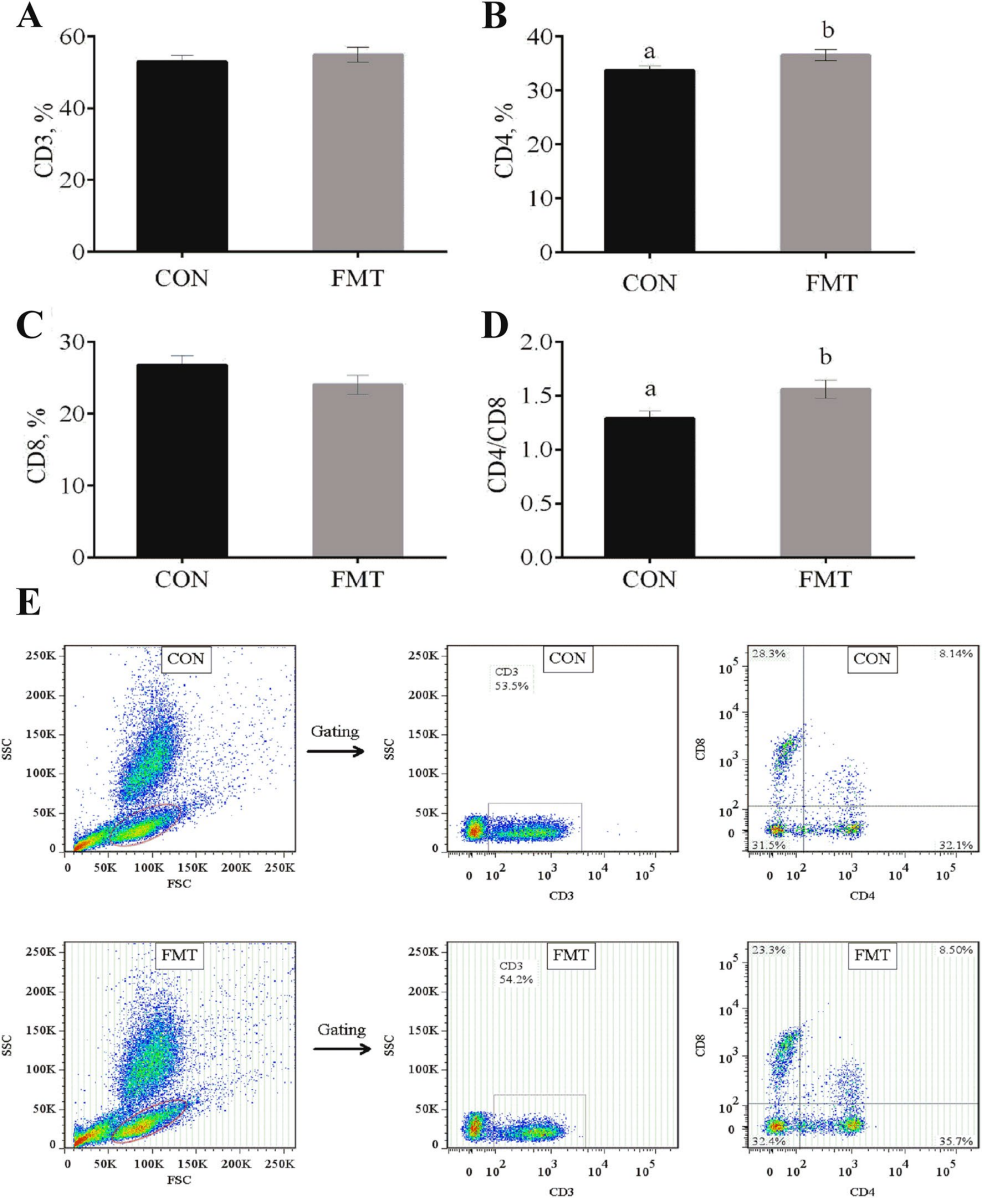
**Analysis of T cell subsets in the peripheral blood of piglets after FMT treatment. A** Capsulized FMT had no significant effect on the CD3^+^ level in the peripheral blood; **B** oral faecal microbial capsule significantly increased the CD4^+^ level; **C** there was no difference in the CD8^+^ level in the peripheral blood between recipient and control piglets; **D** the oral faecal microbial capsule significantly increased the CD4^+^/CD8^+^ ratio; **E** Dot plots of isolated immune cells in the peripheral blood, gated for live cell analysis. Representative flow cytometry data for T cells (CD3^+^ cells, CD3^+^ CD4^+^ cells, CD3^+^ CD8^+^ cells) in the peripheral blood. The data are expressed as the mean ± SEM. a, b represents significant differences between two groups (*n* = 12 per group for **A**–**E**).

The effect of the faecal microbial capsule on the lymphocyte subpopulation in the peripheral blood of weaned piglets is shown in Figure 2. The oral faecal microbial capsule significantly increased the CD4^+^ level (Figure 2B) and CD4^+^/CD8^+^ ratio (Figure 2D) in the peripheral blood (*P* < 0.05), while there were no differences in the CD3^+^ level (Figure 2A) and CD8^+^ level (Figure 2C) ratios in the peripheral blood between recipient and control piglets (*P* > 0.05).

### Gene expression in colon tissue

The mRNA expression measurements of various pattern recognition receptors and associated molecules in the colonic tissues of the recipients are summarized in Table 3. The administration of capsulized FMT significantly decreased TLR2, TLR8, and NF-κB expression compared with that in the control (*P* < 0.05), whereas FMT treatment significantly elevated NOD1 expression in the recipient group compared with that in the control group (*P* < 0.05). In addition, the relative gene expression of tight junction proteins and nitric oxide synthase in the colon tissue are shown in Table 4. Compared with the control group, FMT significantly promoted the expression of ZO-1 (*P* < 0.05), but there were also no significant differences in the mRNA expression levels of occludin and claudin-1. However, after treatment with FMT, the expression of inducible nitric oxide synthases (iNOS) was significantly inhibited (*P* < 0.05).

**Table 3:** Effects of faecal microbial capsules on the expression of genes in the colon of piglets

Items	FMT treatment		SEM	*P* value
	CON	FMT		
TLR2	1.00^b^	0.48^a^	0.011	0.014
TLR3	1.00	2.38	0.443	0.121
TLR4	1.00	1.12	0.258	0.826
TLR7	1.00	0.92	0.193	0.844
TLR8	1.00^b^	0.53^a^	0.103	0.019
TLR9	1.00	0.67	0.142	0.251
NOD1	1.00^a^	1.99^b^	0.248	0.042
NOD2	1.00	1.50	0.256	0.334
NF-κB	1.00^b^	0.32^a^	0.166	0.038
MyD88	1.00	0.76	0.118	0.313

*n* = 14. CON: control group, FMT: faecal microbial transplantation group, TLR: toll-like receptor, NOD: nucleotide binding oligomerization domain.

^a,b^ In the same row, values with different letter superscripts indicate a significant difference.

**Table 4:** Expression of genes related to tight junction proteins and nitric oxide synthase in the colon of piglets

Items	FMT treatment		SEM	*P* value
	CON	FMT		
ZO-1	1.00^a^	1.56^b^	0.110	< 0.01
Occludin	1.00	1.12	0.134	0.687
Claudin-1	1.00	1.28	0.165	0.397
iNOS	1.00^b^	0.55^a^	0.108	0.034
eNOS	1.00	1.19	0.141	0.506

*n* = 14. CON: control group, FMT: faecal microbial transplantation group, CON = piglets in the CON group were fed empty capsules, iNOS: inducible nitric oxide synthases, eNOS: endothelial nitric oxide synthases.

^a,b^ In the same row, values with different letter superscripts indicate significant differences.

## 16S rRNA analysis of gut microbiota

**Figure 3:**
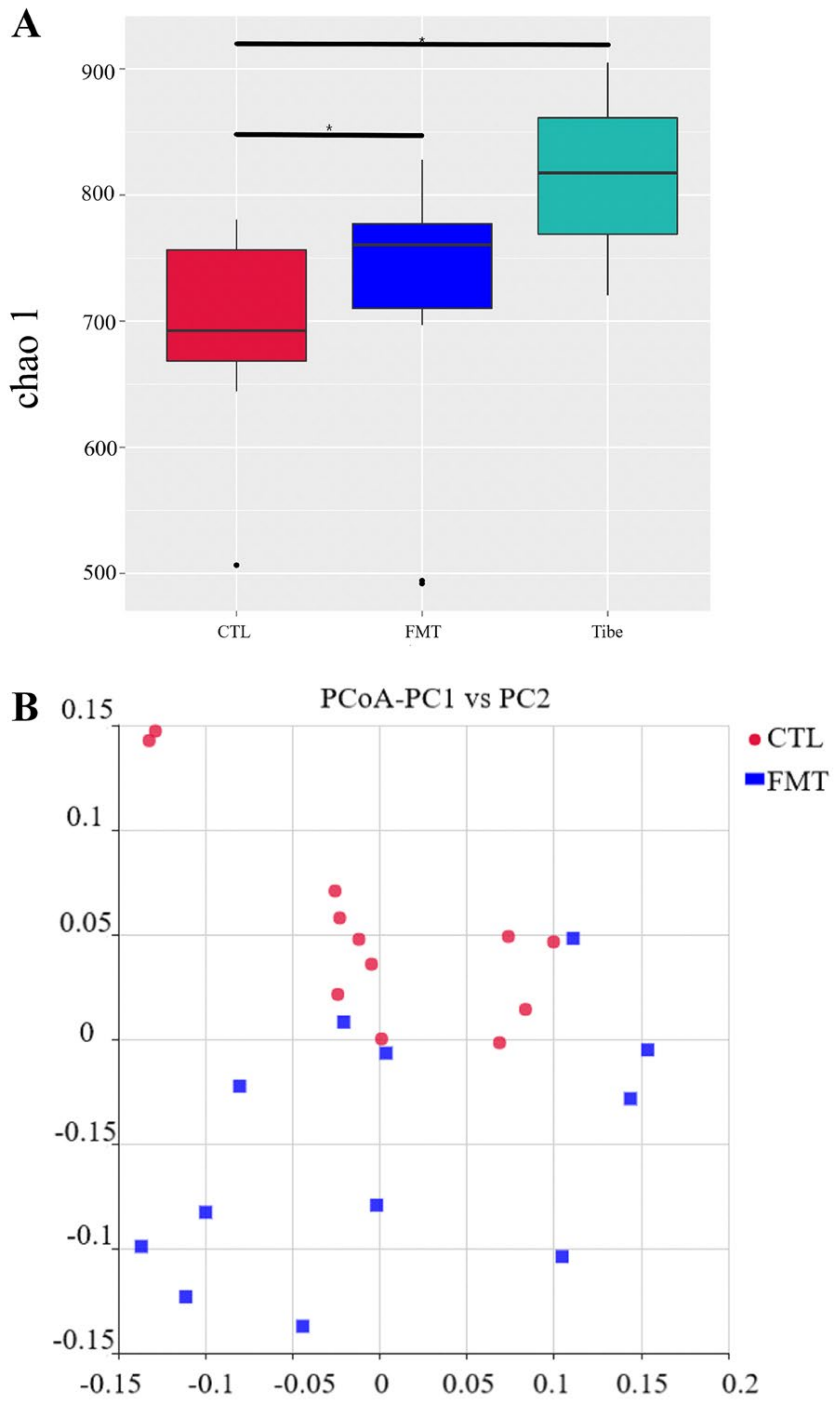
**Effects of capsulized FMT on the alpha diversity and structure of gut microbiota in piglets. A** Chao 1 index calculated after rarefying to an equal number of sequence reads for all samples. The Chao 1 index was increased after FMT treatment; **B** The PCoA score based on weighted UniFrac metrics was significantly different in both groups. The data are expressed as the mean ± SD for (**A**), *P < 0.05. Statistically significant differences were assessed through AMOVA analysis for (**B**) *P < 0.001. In (**A**, **B**), CTL: control group (*n* = 12). FMT: faecal microbiota transplantation (*n* = 12).

The rarefaction curve of observed OTUs plateaued with current sequencing (Additional file 5). The OTU number and alpha diversity index of gut microbiota in piglets are presented in Additional file 6. There was no significant difference in OTU number or Shannon index between the control group and FMT group (*P *> 0.05). However, the Chao 1 indexes were higher in the Tibe group than in the control group (Figure 3A), and FMT treatment significantly increased the Chao 1 index compared with that in the control group (*P* < 0.05). Moreover, a Venn diagram of the three groups revealed that 706 OTUs overlapped among the groups; 960 OTUs were present in the control group and FMT group (Additional file 7). PCoA showed that the gut microbiota in the control group were separated from the FMT group (Figure 3B, Additional file 8), and there was a significant difference in the structure of the gut microbiota (Additional file 9).

**Figure 4:**
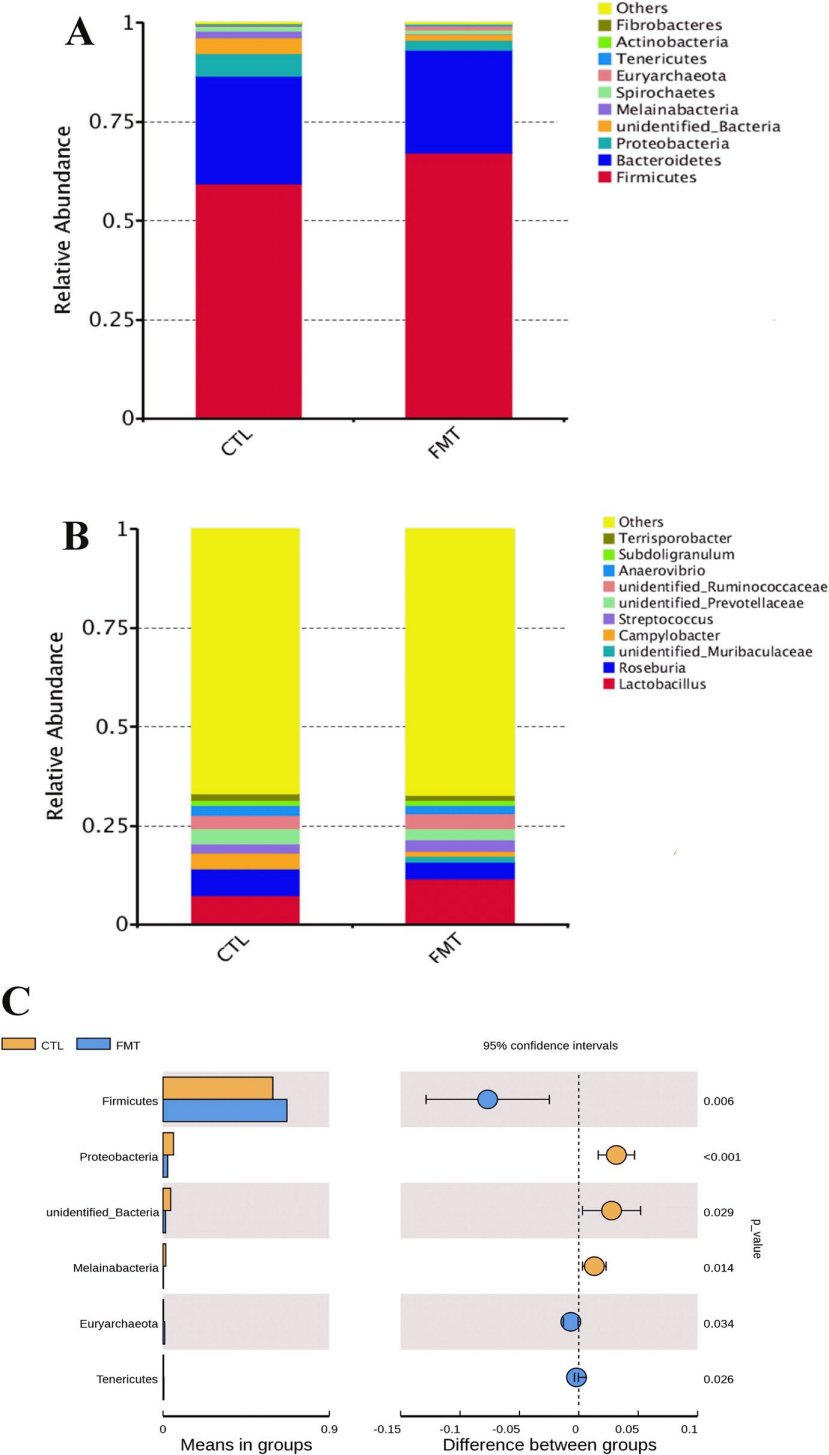
**16S rRNA gene analysis revealed the colonic bacterial composition of piglets. A** Relative abundance of microbiota at the phylum level. **B** Relative abundance of microbiota at the genus level. **C** Differences in the relative abundance of microbiota at the phylum level between the CTL group and FMT group. CTL: control group (*n* = 12). FMT: faecal microbiota transplantation (*n* = 12).

**Figure 5:**
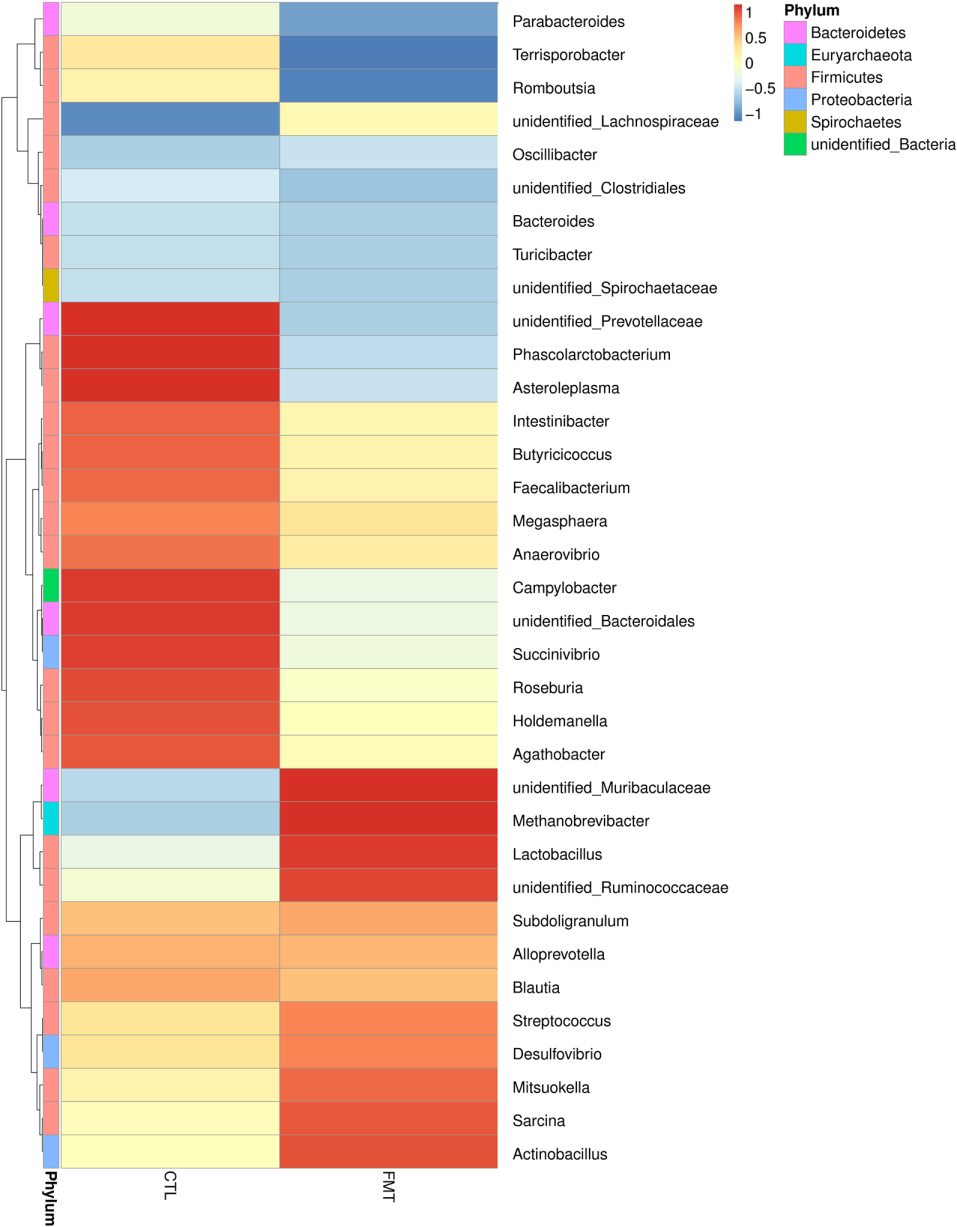
**The distribution of luminal bacteria in the colonic digesta of piglets.** Taxonomic profiles of the microbial communities of the colon were analysed from genera. Pigs with the highest and lowest bacterial levels are red and blue, respectively. CTL: control group (*n* = 12). FMT: faecal microbiota transplantation (*n* = 12).

Histograms illustrate the gut microbiota composition and show the differences in the relative abundance of major gut bacteria at the phylum and genus levels. At the phylum level (Figure 4A), the most abundant phyla in all samples were *Firmicutes*, *Bacteroidetes*, and *Proteobacteria,* which accounted for 92–96% and had the absolute superiority in the gut microbial composition of piglets. FMT treatment significantly increased the relative abundance of *Firmicutes*, *Euryarchaeota*, and *Tenericutes* (*P* < 0.05) and significantly decreased the relative abundance of *Proteobacteria* and *Melainabacteria* compared with the control group (Figure 4C) (*P* < 0.05). The relative abundance of gut microbial composition in the genus family is shown in Additional file 10. The relative abundance of gut microbial composition at the genus level is shown in Figure 4B. Taxonomic profiles and the differences in the microbial communities in the colon were analysed from the genera, and Figure 5 and Table 5 are shown. Compared with the control group, the relative abundance of *Campylobacter* in recipient piglets was significantly decreased (*P* < 0.05), while the relative abundance of *Lactobacillus* and *Methanobrevibacter* in recipient piglets was significantly increased (*P* < 0.05).

**Table 5:** Relative abundance of the most representative genus in the colonic microbiota of weaned piglets

Items	FMT treatment		SEM	*P* value
	CON	FMT		
*Lactobacillus*	0.138^a^	0.712^b^	0.018	0.013
*Campylobacter*	0.042^b^	0.012^a^	0.006	0.042
*Methanobrevibacter*	0.004^a^	0.010^b^	0.001	0.026

Metastats analysis was used to identify the significantly differentially abundant species.

*n* = 12 (values are the means of 12 replicates per treatment, the same below). CON: control group, FMT: faecal microbial transplantation group.

^a,b^ In the same row, values with different letter superscripts indicate significant differences.

## Discussion

The process of weaning is linked to modifications of the intestinal microbiota, which could be related to the aetiology of post-weaning diarrhoea and enteric infections in piglets [9, 26]. However, previous research has established that FMT targets the host to confer diarrhoea resistance in weaned piglets, suggesting the preventative effect of FMT in gastrointestinal tract disorders [27]. Furthermore, the efficacy of FMT in piglet diarrhoea prevention also helps to raise the possibility of using intestinal microbiota resources from native pig breeds [28, 29]. In the present study, crossbred DLY piglets were selected to receive capsulized faecal microbiota transplantation from healthy Tibetan pigs. Our results demonstrated that the diarrhoea incidence and diarrhoea indexes were significantly decreased in FMT-treated piglets, which was in line with previous studies [27, 30]. During the weaning transition, piglets are susceptible to diarrhoea, which has been associated with a disrupted state of the microbiota and the immature immune system [31, 32]. Khoruts has noted that host phenotypes (diarrhoea resistance) can be altered by FMT in mammals, indicating potential links between intestinal microbiota and gut health [33]. To further uncover the underlying mechanism of FMT and verify the gut microbes that confer anti-diarrhoea effects, we used 16S rRNA sequencing to facilitate the investigation of alterations in intestinal microbial composition by FMT in weaning piglets.

Our results showed that FMT treatment altered the alpha diversity of the intestinal microbiota community based on a higher Chao 1 index compared with that of control-treated piglets. Moreover, PCoA analysis showed significant distances between both groups, consistent with a previous study revealing that FMT treatment could change the beta diversity of the gut microbiota [28]. Furthermore, the distance in the PCoA analysis was separated between the control group and FMT group, which indicated that the transplantation of the faecal microbiota capsule modulates the intestinal microbiota structure of recipient piglets. Our study was consistent with previous investigations [34, 35], which indicated that FMT can effectively change the structure and diversity of the intestinal microbiota. On the other hand, the results implied that the instability of the gut microbiota of weaning pigs caused by weaning stress was likely restored by introducing the Tibetan pig’s intestinal microbiota, which was similar to previous research findings [36]. The present study showed that the relative abundance of *Firmicutes* in the colon of recipient piglets was significantly increased. *Firmicutes* produce short-chain fatty acids known to maintain intestinal health by suppressing inflammation and providing energy to enterocytes [37]. Thus far, common findings include the depletion of the phylum *Firmicutes*, which impacts the exacerbation of intestinal inflammation [38]. Interestingly, our FMT group showed intact morphology and a lower histological damage score, indicating that capsulized faecal microbiota effectively mitigated weaning-induced inflammation and influenced the mucosal immune response. The most predominant species of methanogens were related to the genus *Methanobrevibacter* in the pig gut ecology [39], which fell within the phylum *Euryarchaeota* [40]. Coincidentally, significant differences in the abundance of *Euryarchaeota* and *Methanobrevibacter* were correlated with apparent crude fibre digestibility in pigs [41]. Other studies showed that transferring faecal microbiota from Tibetan pigs into DLY piglet recipients significantly restructured the gut microbiota, and the physiological differences induced by the intake of coarse feedstuff might transmit to the piglets [13, 28], which allowed them to adapt to dietary changes and improve diarrhoea after early weaning [42]. A broad pattern of dysbiosis in post-weaning diarrhoea of pigs has been noted, characterized by a reduction in *Bacteroidetes*, a loss of microbial diversity within the *Firmicutes* phylum and an increased proportion of *Proteobacteria* [43]. Our observations suggested that FMT could be used therapeutically to correct these abnormalities and decrease gut inflammation.

*Lactobacillus* are major players in disease prevention, and their abrupt reduction during the weaning transition contributes to the increase in disease risk [44]. Our study showed that *Lactobacillus* in recipient piglets was increased. A recent study highlighted that some of the microbiota, including *Lactobacillus*, can induce immunomodulatory-like regulatory T cells in both the small intestinal and colonic lamina propria [45]. Our study found that the administration of FMT capsules to piglets significantly attenuated the decrease in CD4^+^ T cells and CD4^+^/CD8^+^ ratio in the peripheral blood. As members of the cellular immune system, CD4^+^ T cells play a pivotal role in protecting animals and humans against pathogens, and CD4^+^ T cells are especially essential to enhance cytokine production [46]. Thus, these results could partially explain the effect of FMT capsule supplementation on immunity maintenance and inflammation control in piglets after weaning. In contrast, piglets in the FMT group displayed a reduced relative abundance of *Campylobacter* compared with that in the control group. The current findings suggest that emerging *Campylobacter* may play a part in the pathogenesis of a subset of these gastrointestinal disorders, such as diarrhoea and other serious gastrointestinal symptoms [47]. Although the underlying mechanisms are not fully understood, the above results suggested that protection of piglets from post-weaning diarrhoea and excessive inflammation by FMT capsules could notably occur by favouring the establishment of bacteria that benefit the host, reducing harmful bacterial growth and adhesion to intestinal mucosa, and stimulating the recipient piglets’ immune system.

Cytokines secreted by mucosal antigen-presenting cells greatly impact the initiation and propagation of intestinal inflammation, as in the maintenance of homeostasis [48]. We further investigated the effect of FMT-derived microbiota on the intestinal cytokine milieu. A recent study showed that therapeutic FMT reduces colonic inflammation and initiates the restoration of intestinal homeostasis through the simultaneous activation of different immune-mediated pathways, ultimately leading to IL-10 production by innate and adaptive immune cells, including CD4^+^ T cells [49]. Consistently, our results showed that colonic mucosa exposed to FMT increases the level of IL-10, a cytokine with broad anti-inflammatory properties, which is an important self-regulatory function of CD4^+^ T lymphocytes [50]. Upon FMT, the secretion of IL-10 and the level of CD4^+^ T cells simultaneously increased, which suggested that variations in the intestinal ecology towards IL-10 inducing microbial communities and the production of tolerogenic IL-10 by colonic mucosa T lymphocyte subsets together resolve the intestinal inflammation of piglets. Furthermore, FMT treatment resulted in up-regulation of another anti-inflammation cytokine (IL-4) and reduction of pro-inflammation cytokines, such as TNF-α and IFN-α, which are important mediators of the formation of colonic inflammation and diarrhoea in piglets. In addition, some inflammatory cytokine genes have binding sites for NF-κB and are regulated by the transcription of these factors [51]. These changes collectively demonstrated that FMT treatment strengthens the gut immune barrier function by increasing some immune factors and reducing the secretion of pro-inflammatory cytokines.

Many microbial products are sensed by pattern recognition receptors, such as TLRs, which are critical for protection against gut inflammation and injury [52]. It has been demonstrated that TLR2 activation leads via a cascade of intermediary steps to NF-κB activation [53]. In our study, variations in the microbial communities in FMT treatment may exert interactions with the intestinal immune system of piglets. The observations exactly showed that the colonic mRNA expressions of TLR2 and NF-κB were both significantly down-regulated in recipient piglets, which suggested that FMT treatment reduced TLR2 responsiveness and induced TLR tolerance and further directly restrained NF-κB translocation into the nucleus to induce pro-inflammatory cytokine gene expression. Evidence from mouse experiments also indicated the important role of TLR2 in the pro-inflammatory state [54]. Therefore, these findings in the present study may provide an explanation for the outcome in which the diarrhoea of the piglets was ameliorated by activating TLR signalling, which prevented excessive intestinal inflammation after FMT treatment. In addition, our results showed that FMT treatment mildly decreased the colonic mRNA expression of TLR8, which suggested a latent link between TLR8 and inflammatory release in response to gut microbiota. However, the exact mechanisms of TLR8 activation in response to the immune recognition of bacterial RNA and inflammatory cytokine production remain to be investigated [55].

One of the symptoms of diarrhoea of weaned piglets is disruption of the intestinal epithelial barrier, which may result in inflammation [56], causing inefficiency at preventing the invasion of bacteria while allowing nutrient absorption. The intestinal epithelial barrier is primarily regulated by a well-organized system of an epithelial junctional complex referred to as the tight junction. ZO-1 is the most important constituent in the structural and functional organization of tight junctions associated with epithelial integrity [57]. From the results of the colonic tissue section, the mRNA expression of ZO-1 demonstrated the significant therapeutic effects of FMT treatment. A related study also reported the possible mechanisms and pointed out that the improvement of intestinal integrity via microbiota transfer might be a potential target for the treatment of diarrhoea in weaned piglets [30]. Intriguingly, a reduction in the expression of iNOS was observed in our study. The nitric oxide (NO) glut released by iNOS directly or indirectly causes damage to target intestinal mucosa tissues, engendering gut inflammation [58]. Previous studies reported that NF-κB directly induces the upregulation of iNOS, which is also involved in intestinal inflammation [59]. Therefore, our results likely obtained additional evidence of an anti-inflammatory effect, namely, down-regulation of the expression of NF-κB expression, which further induced a decline in iNOS synthesis and alleviated the extent of inflammatory.

Overall, capsule FMT ameliorates post-weaning diarrhoea and decreases the F/G ratio in piglets. These effects might be attributed to modulated colonic microbiota, improved colonic morphology, gut barrier function, and innate immunity in the present study.

## Supplementary information

**Additional file 1. Ingredients and nutrient composition of the basal diet (as-fed basis).**^1^ CP, crude protein; SID, standard ileal digestible.^2^ Vitamin premix provided the following per kg of diet: VA, 6000 IU; VD3, 400 IU; VE, 10 IU; VK3, 2 mg; VB1, 0.8 mg; VB2, 6.4 mg; VB6, 2.4 mg; VB12, 12 µg; folic acid, 0.2 mg; nicotinic acid, 14 mg; D-pantothenic acid, 10 mg.^3^ Mineral premixes provided the following per kg of diets: Fe (ferrous sulfate) 100 mg, Cu (copper sulfate) 6 mg, Mn 4 mg, Zn (zinc sulfate) 100 mg, and I (potassium iodide) 0.14 mg

**Additional file 2. Histological damage score.**

**Additional file 3. Real-time PCR primers and conditions.**

**Additional file 4. The diarrhoea score of each piglet.** Stool consistency (0, normal; 1, pasty; 2, semiliquid; and 3, liquid) was reported daily; pigs showing scores of 2 or 3 were considered to have diarrhoea; d1-21 represent experimental days; CON, control group; FMT, faecal microbial transplantation group.

**Additional file 5. Rarefaction curves of observed OTUs.** CTL, control group; FMT, faecal microbial transplantation group; Tibe, Tibetan pig group.

**Additional file 6. The OTU numbers and alpha diversity indexes of colonic microflora in weaned piglets**^**1**^. ^1^*n* = 12. CON, control group; FMT, faecal microbial transplantation group; CON = piglets in the CON group were fed vacant capsules; FMT = piglets in FMT group were fed faecal microbial capsules.

**Additional file 7. Venn diagram indicated the differential numbers of OTUs in both groups.** CTL, control group (*n* = 12). FMT, faecal microbiota transplantation (*n* = 12). Tibe, Tibetan pig group (*n* = 3).

**Additional file 8. PCoA analysis of pigs based on weighted UniFrac metrics.** CTL, control group (*n* = 12). FMT, faecal microbiota transplantation (*n* = 12). Tibe, Tibetan pig group (*n* = 3).

**Additional file 9. PCoA visualization with statistical tests of pigs based on weighted UniFrac metrics.** CTL, control group (*n* = 12). FMT, faecal microbiota transplantation (*n* = 12).

**Additional file 10. 16S rRNA gene analysis revealed relative abundance of microbiota in the level of family.** CTL, control group (*n* = 12). FMT, faecal microbiota transplantation (*n* = 12).

## Data Availability

The datasets generated and/or analysed during the current study are available from the corresponding authors on reasonable request. The raw sequencing data are available in the National Center for Biotechnology Information (NCBI) GEO repository under accession number GSE137470 [60].

## Abbreviations

FMT: faecal microbiota transplantation
F/G: ratio feed to gain
TLR: toll-like receptor
iNOS: inducible nitric oxide synthases
eNOS: endothelial nitric oxide synthases
DLY: Duroc × Landrace × Yorkshire
NRC: national research council
BW: body weight
ADG: average weight gain
ADFI: average daily feed intake
OTU: operational taxonomic unit
PCoA: principal coordinates analysis
NOD: nucleotide binding oligomerization domain
