# Supplementary material for: Capsulized faecal microbiota transplantation ameliorates post-weaning diarrhoea by modulating the gut microbiota in piglets
Source: Vet Res. 2020 Apr 16;51:55. doi: 10.1186/s13567-020-00779-9 (PMC7164362; doi:10.1186/s13567-020-00779-9)
Supplement: Supplementary file 1 — Additional file 1. Ingredients and nutrient composition of the basal diet (as-fed basis).1 CP, crude protein; SID, standard ileal digestible.2 Vitamin premix provided the following per kg of diet: VA, 6000 IU; VD3, 400 IU; VE, 10 IU; VK3, 2 mg; VB1, 0.8 mg; VB2, 6.4 mg; VB6, 2.4 mg; VB12, 12 µg; folic acid, 0.2 mg; nicotinic acid, 14 mg; D-pantothenic acid, 10 mg.3 Mineral premixes provided the following per kg of diets: Fe (ferrous sulfate) 100 mg, Cu (copper sulfate) 6 mg, Mn 4 mg, Zn (zinc sulfate) 100 mg, and I (potassium iodide) 0.14 mg [file 13567_2020_779_MOESM1_ESM.docx]

**Additional file 1**. **Ingredients and nutrient composition of the basal diet^1^(as-fed basis)**

| Ingredients | % | |
| --- | --- | --- |
| Corn (CP 7.8%) | 31.60 | |
| Extruded corn | 31.61 | |
| Soybean meal (CP 46%) | 10.00 | |
| Extruded Soybean | 5.00 | |
| Fish meal (CP 62.5%) | 3.50 | |
| Plasma protein powder | 1.90 | |
| Whey powder | 5.00 | |
| Soybean protein concentrate | 2.50 | |
| Soybean oil | 2.25 | |
| Sucrose | 3.00 | |
| Limestone | 0.88 | |
| Dicalcium phosphate | 0.39 | |
| Salt | 0.20 | |
| L-Lys·HCl | 0.70 | |
| DL-Met | 0.34 | |
| L-Thr | 0.29 | |
| L-Trp | 0.14 | |
| L-Val | 0.25 | |
| Chloride choline | 0.10 | |
| Vitamin premix^2^ | 0.05 | |
| Mineral premix^3^ | 0.30 | |
| Total | 100.00 | |
| **Nutrient levels** | |  |
| DE, Mcal/kg | 3.54 | |
| CP | 18.05 | |
| Calcium | 0.79 | |
| Total phosphorus | 0.54 | |
| SID Lysine | 1.43 | |
| SID Methionine + Cysteine | 0.83 | |

^1^ CP, crude protein; SID, standard ileal digestible.

^2^ Vitamin premix provided the following per kg of diets: VA, 6000 IU; VD_3_, 400 IU; VE, 10 IU; VK_3_, 2 mg; VB_1_, 0.8 mg; VB_2_, 6.4 mg; VB_6_, 2.4 mg; VB_12_, 12 µg; folic acid, 0.2 mg; nicotinic acid, 14 mg; *D-*pantothenic acid, 10 mg.

^3^ Mineral premixes provided the following per kg of diets: Fe (ferrous sulfate) 100 mg, Cu (copper sulfate) 6 mg, Mn 4 mg, Zn (zinc sulfate) 100 mg, I (potassium iodide) 0.14 mg.
