# Supplementary material for: Capsulized faecal microbiota transplantation ameliorates post-weaning diarrhoea by modulating the gut microbiota in piglets
Source: Vet Res. 2020 Apr 16;51:55. doi: 10.1186/s13567-020-00779-9 (PMC7164362; doi:10.1186/s13567-020-00779-9)
Supplement: Supplementary file 2 — Additional file 2. Histological damage score. [file 13567_2020_779_MOESM2_ESM.docx]

**Additional file 2** **Histological damage score**

| Score | Inflammation severity | Inflammation extent | Bleeding | Crypt damage |
| --- | --- | --- | --- | --- |
| 0 | None | None | None | None |
| 1 | Mild | Mucosa | Mild | Basal 1/3 damage |
| 2 | Moderate | Submucosa | Moderate | Basal 2/3 damage |
| 3 | Severe | Transmural | Severe | Crypt lost |
