# Supplementary material for: Capsulized faecal microbiota transplantation ameliorates post-weaning diarrhoea by modulating the gut microbiota in piglets
Source: Vet Res. 2020 Apr 16;51:55. doi: 10.1186/s13567-020-00779-9 (PMC7164362; doi:10.1186/s13567-020-00779-9)
Supplement: Supplementary file 3 — Additional file 3. Real-time PCR primers and conditions. [file 13567_2020_779_MOESM3_ESM.docx]

**Additional file 3** **Real-time PCR primers and conditions**

| Gene | GenBank accession | Primer sequences (5’ to 3’) | Size (bp) | Annealing (℃) |
| --- | --- | --- | --- | --- |
| TLR2 | AY392087.1 | CGGCTTCCAAGGATGGA | 114 | 60 |
|  |  | CAATCCCCAAGACCCAT |  |  |
| TLR3 | NM_001097444.1 | TGGAAAAAGGAATGGCCAGC | 218 | 60 |
|  |  | ACAAGGCAAACTCCTGCTCA |  |  |
| TLR4 | GQ304754 | TTACAGAAGCTGGTTGCCGT | 152 | 60 |
|  |  | TCCAGGTTGGGCAGGTTAGA |  |  |
| TLR7 | EF583901.1 | CAATGGTCCCTGAGCGTTT G | 152 | 60 |
|  |  | AGCCTGGTTGAAGACAGCAG |  |  |
| TLR8 | NM_214187.1 | AGAGCTGCTAATTGGTGCCTT | 214 | 60 |
|  |  | AGGCAGGTCAGGAGCAAAAA |  |  |
| TLR9 | NM_213958.1 | CCAGCCAGACCCTTTGGAGA | 174 | 60 |
|  |  | GGAGAGTAAGGAGAGGCTGGT |  |  |
| NOD1 | NM_001114277.1 | TCAACACCGATCCAGTGAGC | 237 | 60 |
|  |  | TGAAAATGGTCTCGCCCTCC |  |  |
| NOD2 | NM_001105295.1 | GTGCCTCCCCTCTAGACTCA | 191 | 60 |
|  |  | ACGAACCAGGAAGCCAAGAG |  |  |
| NF-κB | DQ834921.1 | TTCTGGACCGCTTGGGTAAC | 120 | 60 |
|  |  | CACCGTTGGGGTGGTTGATA |  |  |
| MyD88 | AB292176.1 | GCTCTTCCTAAACGTGCGGA | 241 | 60 |
|  |  | TCGGCAGTCCTCTTCAATGC |  |  |
| ZO-1 | XM_005659811 | CTGAGGGAATTGGGCAGGAA | 105 | 60 |
|  |  | TCACCAAAGGACTCAGCAGG |  |  |
| Occludin | NM_001163647.2 | CAGGTGCACCCTCCAGATTG | 110 | 60 |
|  |  | GGACTTTCAAGAGGCCTGGAT |  |  |
| CLDN | FJ873109.1 | GCCACAGCAAGGTATGGTAAC | 158 | 61 |
|  |  | AGTAGGGCACCTCCCAGAAG |  |  |
| iNOS | NM_001143690.1 | GAGCCCAGAGGGCTTTATCA | 126 | 59 |
|  |  | TTCTTTGCTGTCTCCGCCAG |  |  |
| eNOS | NM_214295.1 | AGGTGGGGAGCATCACCTAT | 175 | 59 |
|  |  | TGGTTGATGAAGTCCCTGGC |  |  |
| β-Actin | NM_001101 | TCTGGCACCACACCTTC | 114 | 57 |
|  |  | TGATCTGGGTCATCTTC |  |  |
