# Supplementary figures and images for: Capsulized faecal microbiota transplantation ameliorates post-weaning diarrhoea by modulating the gut microbiota in piglets
Source: Vet Res. 2020 Apr 16;51:55. doi: 10.1186/s13567-020-00779-9 (PMC7164362; doi:10.1186/s13567-020-00779-9)

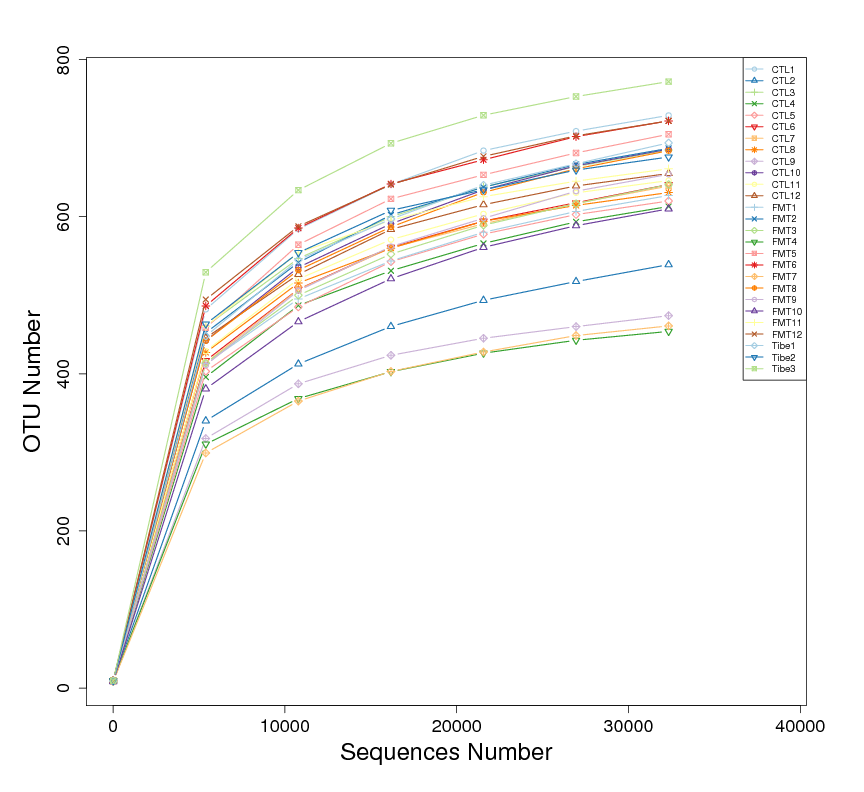

Supplement: Supplementary file 5 — Additional file 5. Rarefaction curves of observed OTUs. CTL, control group; FMT, faecal microbial transplantation group; Tibe, Tibetan pig group. [file 13567_2020_779_MOESM5_ESM.png]

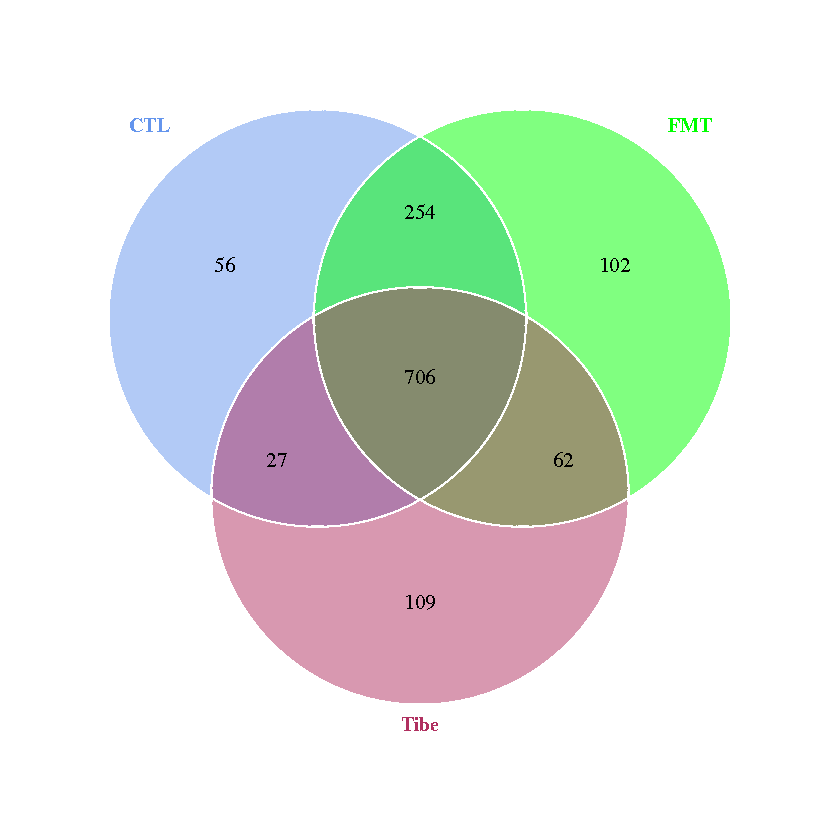

Supplement: Supplementary file 7 — Additional file 7. Venn diagram indicated the differential numbers of OTUs in both groups. CTL, control group (n = 12). FMT, faecal microbiota transplantation (n = 12). Tibe, Tibetan pig group (n = 3). [file 13567_2020_779_MOESM7_ESM.png]

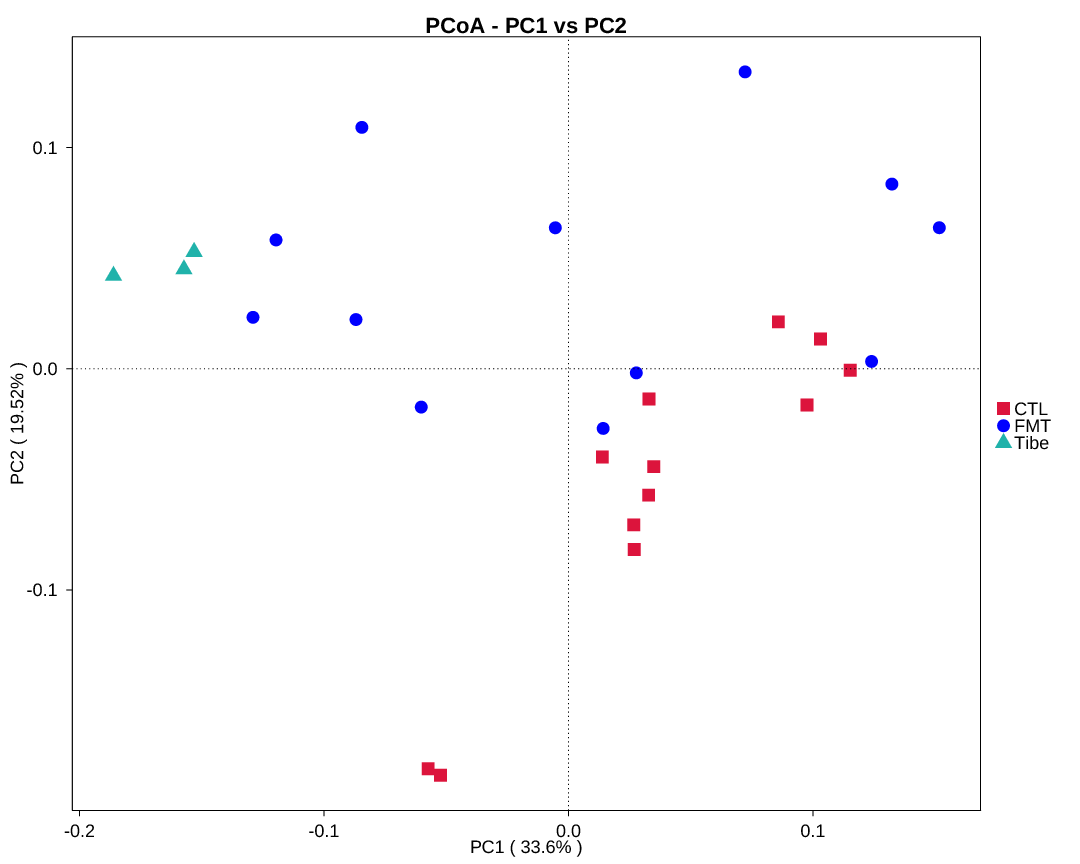

Supplement: Supplementary file 8 — Additional file 8. PCoA analysis of pigs based on weighted UniFrac metrics. CTL, control group (n = 12). FMT, faecal microbiota transplantation (n = 12). Tibe, Tibetan pig group (n = 3). [file 13567_2020_779_MOESM8_ESM.png]

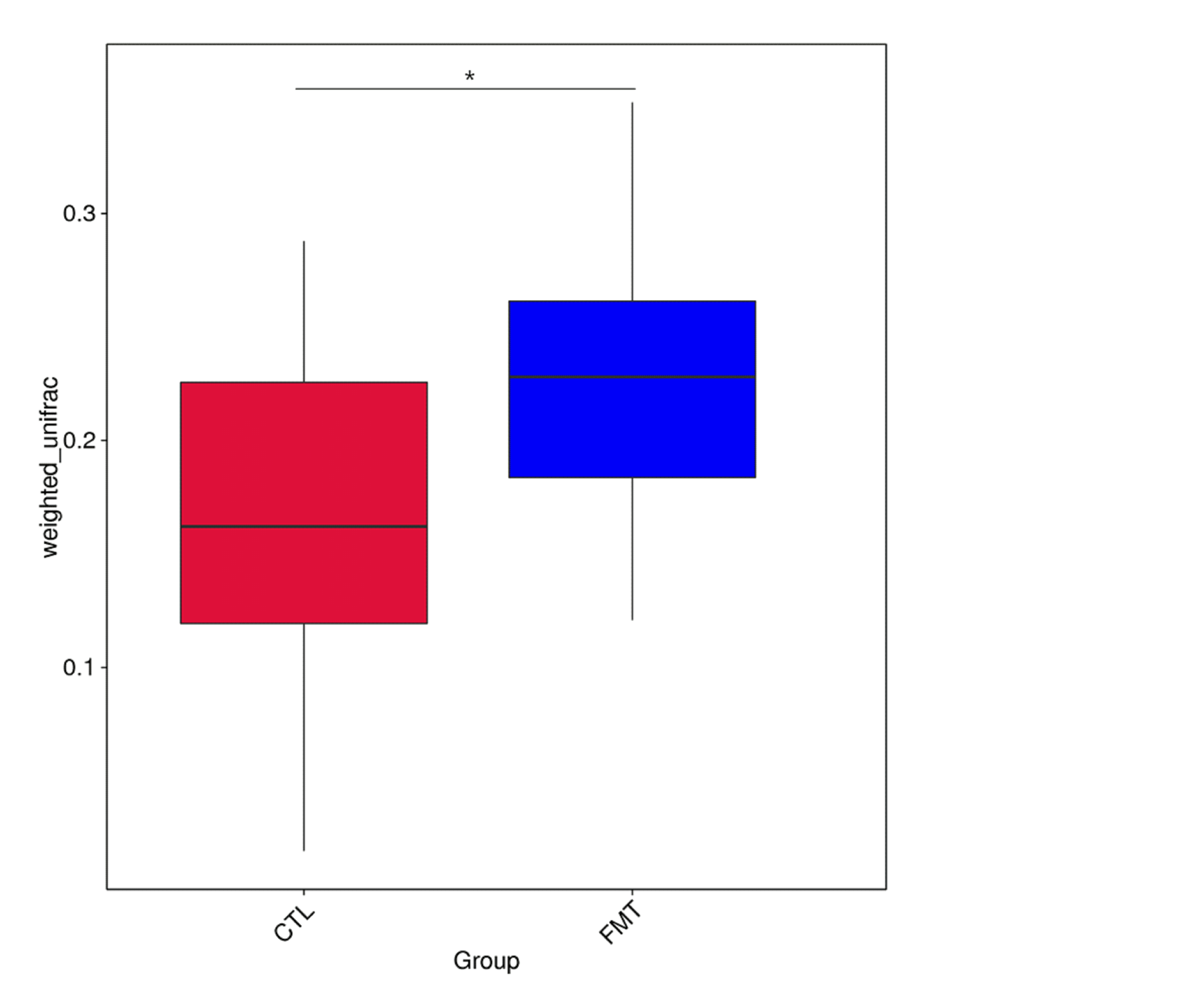

Supplement: Supplementary file 9 — Additional file 9. PCoA visualization with statistical tests of pigs based on weighted UniFrac metrics. CTL, control group (n = 12). FMT, faecal microbiota transplantation (n = 12). [file 13567_2020_779_MOESM9_ESM.png]

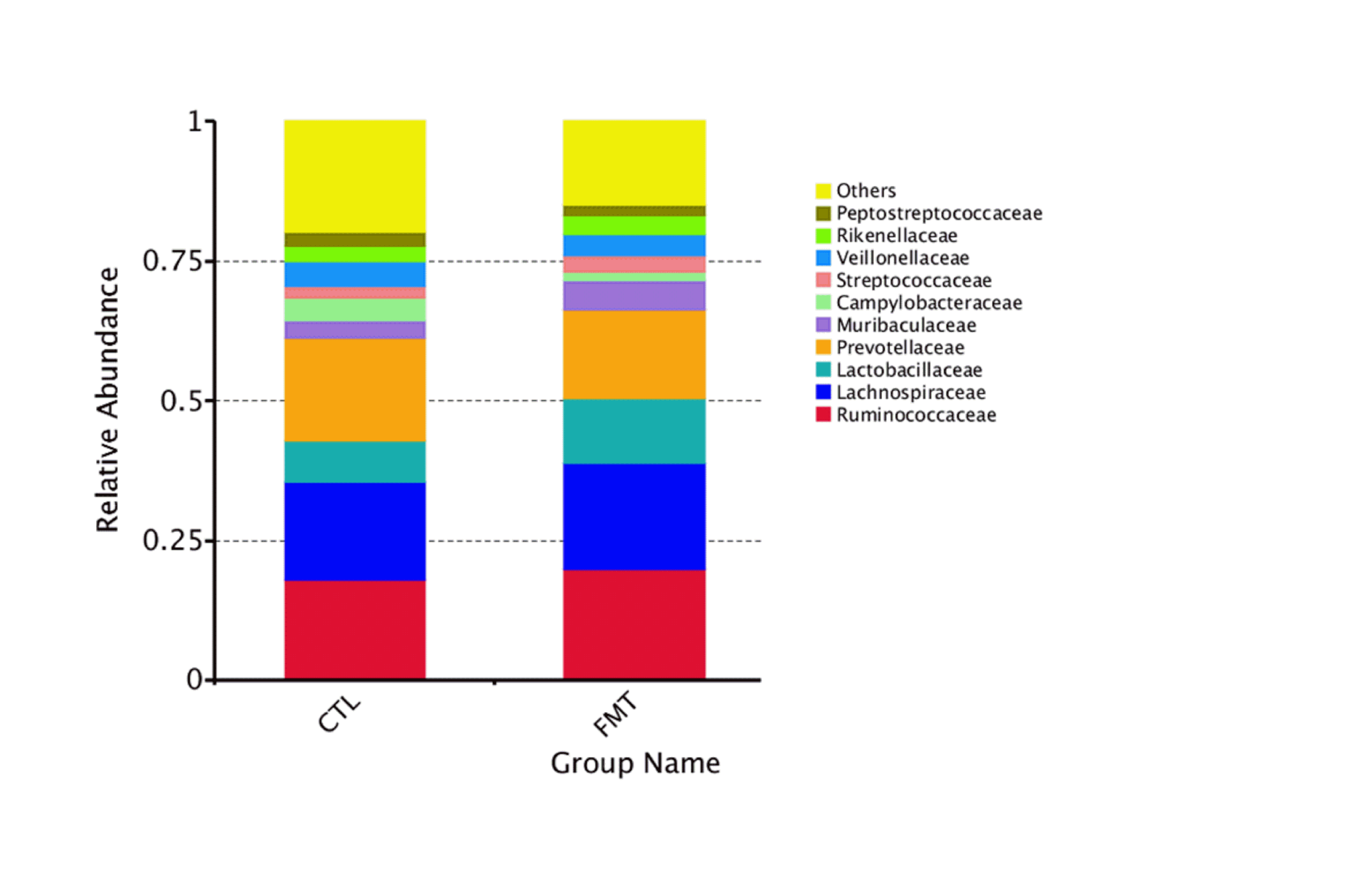

Supplement: Supplementary file 10 — Additional file 10. 16S rRNA gene analysis revealed relative abundance of microbiota in the level of family. CTL, control group (n = 12). FMT, faecal microbiota transplantation (n = 12). [file 13567_2020_779_MOESM10_ESM.png]
