# Supplementary material for: Capsulized faecal microbiota transplantation ameliorates post-weaning diarrhoea by modulating the gut microbiota in piglets
Source: Vet Res. 2020 Apr 16;51:55. doi: 10.1186/s13567-020-00779-9 (PMC7164362; doi:10.1186/s13567-020-00779-9)
Supplement: Supplementary file 6 — Additional file 6. The OTU numbers and alpha diversity indexes of colonic microflora in weaned piglets1. 1n = 12. CON, control group; FMT, faecal microbial transplantation group; CON = piglets in the CON group were fed vacant capsules; FMT = piglets in FMT group were fed faecal microbial capsules. [file 13567_2020_779_MOESM6_ESM.docx]

**Additional file 6 The OUT numbers and** **alpha diversity indexes of colonic microflora in weaned piglets^1^**

| Items | FMT treatment | | SEM | *P* value |
| --- | --- | --- | --- | --- |
|  | CON | FMT |  |  |
| OTU | 686.33 | 734.33 | 13.345 | 0.071 |
| Chao 1 | 696.32^a^ | 754.18^b^ | 12.427 | 0.015 |
| Shannon | 6.91 | 6.80 | 0.084 | 0.550 |

^1^*n* = 12. CON, control group; FMT, fecal microbial transplantation group; CON = piglets in the CON group were fed vacant capsule; FMT = piglets in FMT group were fed fecal microbial capsule.
